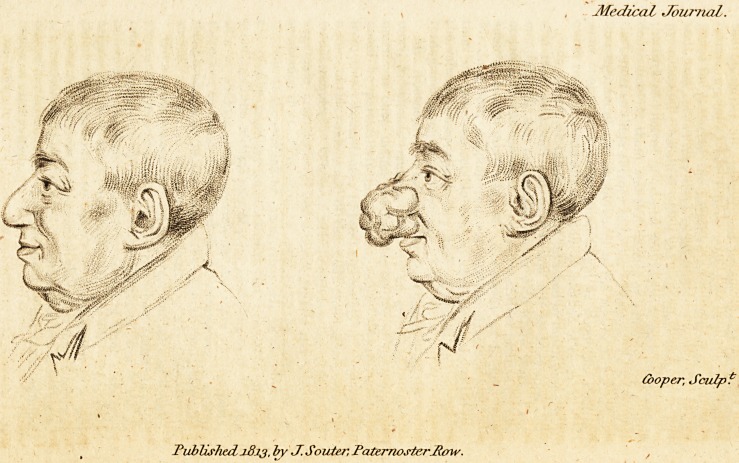# Mr. Barlow's Case of Morbid Tumor on the Nose

**Published:** 1813-04

**Authors:** James Barlow

**Affiliations:** Blackburn, Lancashire


*To the Editors of the Medical and Physical Journal.*

(With an Engraving.)

GENTLEMEN,

BY inserting the following Case of Morbid Tumor on the Nose, and the preliminary remarks connected therewith, in the next Number of the Medical and Physical Journal, you will much oblige

*Blackburn, Lancashire,* Your obedient Servant,

*March* 2, 1813. JAMES BARLOW.

I have been induced, by the urgent intreaty of several persons, to lay the following case before the public, but more particularly by Mr. Blakey himself, whose character for liberality of sentiment, and usefulness in the exercise of his office, as schoolmaster here, is justly appreciated, and widely extended. Were I urged to give publicity to this almost unexampled production of nature, by no other motive

 than

Cooper, Sculp.[t]

Published 1813, by J. Souter, Paternoster Row.

than its peculiar character, and the success resulting from the operation, the professional reader would pardon any further apology; but I have moreover to crave his indulgence for prefacing the narrative with a few analogical observations, which may serve in some degree to explain those preternatural gibbosities which are sometimes observable on the bark and leaves of plants, and likewise shew the coincidence betwixt them and that species of tumor which forms the sequel to these cursory remarks. Perhaps, by a minute investigation of the exuberances in the vegetable kingdom, some comparative information might be acquired, relative either to their mode of formation, or method of removal.

The subject of tumors is yet involved in much obscurity, and their structure and designation are so diversified and complicated, that no modern author with whose works I am acquainted, has ventured to publish a distinct treatise on the subject, except my friend, Mr. Abernethy, of London, and Mr. John Bell, of Edinburgh. It is to the elaborate productions of these respectable writers, that we are indebted for the valuable observations they have advanced on this interesting and momentous subject.

Mr. Abernethy, in his work on the Classification of Tumors, judiciously remarks, that, "if an arrangement of tumors was once made, so that the history of each species could be particularly marked, we might perhaps be able, from this circumstance, to form a probable opinion of the nature of this tumor, and of the mode of treatment which it would require." Whoever strictly surveys the animal and vegetable creation, will find a much greater and nearer resemblance than at first would appear to be the case; for, if we look at the stamina of plants, they will be found to consist of fine capillary tubes, which run parallel with each other, and the cortical part of trees is double, as well as the skin of animals; and, on viewing the more interior parts, there will be found distributed the parenchymous, as well as the fibrous, and in the centre the pith or medullary substance, as also air-vessels, lymphatics, and lacteals.

On a retrospective view of the great difficulty of exactly ascertaining the respective boundaries which appear to separate the animal from the vegetable kingdom, and of the similitude of the structure and functions of their respective organs destined for nourishment and growth, from their primordial state to the period of old age and decay, it will appear evident from such research, that both these kingdoms are influenced by similar laws; and that Nature, in the exercise of her agency, has been governed by the same dignified model in each system.

It is a fact generally known to physiologists, that the laws and ordinances in maintaining and supporting the animal and vegetable actions are supported and carried on by a process of living and active vessels, not very dissimilar from each other; consequently, we occasionally witness monstrosities and deformities in each kingdom, and the different distinct states of infancy, maturity, and decay, are marked by nature, and become as inevitable in plants, and proceed with as much seeming regularity, as in the most perfect animals. A plant can no more live without leaves and air-vessels, than an animal without cuticle and lungs. Is not then the motion of the sap in plants like that of the blood in animals, produced chiefly by the action of the air combined with external heat?

The deformities we behold in vegetables can frequently be traced to some accident or chemical fault in the soil; and the cause of those preternatural deviations from nature belonging to the human species, may not unaptly be exemplified by the analogy of the vegetable kingdom. We have many cogent facts to corroborate this opinion, which shew that the fibres and vessels of animals and vegetables are possessed of a wonderful capacity of expanding and inosculating themselves into one another; and of carrying on a reciprocal circulation, and becoming endued with the power of either restoring lost parts, or causing supernumerary ones; to this degree of plenitude in the vascular system may be ascribed the production of tumors on animals, and the like deformities on plants; and, by a similar process of the functions of vegetable action, buds and branches or shoots of one tree may be implanted into another by grafting or inoculation, the fibres of which are insinuated and incorporated into those of the original stock, so that they are renovated into one being. In like manner fractured bones are united, and the sutures of the skull, and epiphysis of bones, become totally obliterated in old age; and every breach, whether in the vegetable or animal system, is repaired by the same inherent process of living inosculating action.

Let us, however, for a moment compare the faculty of regeneration and self-preservation, which vegetables possess with those of man, and the superiority of the former will be easily perceived to overrate the latter. It is manifestly evident, that vegetables suffer great violence and destruction of parts with apparent impunity; this inherent power which they inherit of cicatrising wounds made in the bark of trees, and restoring lost branches, is so inevitable and wonderful, as to induce us to believe, that there is scarcely any bounds prescribed by nature to this living and almost inexhaustible

 faculty.

faculty. But the human species, though at the head of the creation, are not indued with such efficient influence to the same extent; for our fabric is, by a wise and immutable law of nature (though concealed from us), so modified and complicated, that the necessary wants to support our present existence, which must be perpetually supplied, together with the depravations of our nature, by which we are subject to many physical evils, unavoidably predispose man to various diseases and limitations of autocracy and self-preservation, to which the vegetable system is wholly exempt: yet the analogy of growth in both is such, that the excrescence noticed on some trees, and the preternatural tumor which is formed on the human body, are produced and generated by a vital action very analogous to each other; for, when a plant is wounded by some external cause, there takes place a copious exudation of lymph from the breach, and, if the wound be not mortal, granulations copiously shoot out from vessels on all sides, and the part becomes healed by a cicatrization similar to what takes place in animals. It is also an inevitable law of nature, that both plants and animals, sooner or later, when their course is finished for which they were destined here, must be subdued by death, and return to dust again.

Little as these crude remarks may appear to possess either of novelty or interest, yet, if we take a glance at the organization of the lower order of the living system of nature, and compare the simple with the more complicated and exalted animals, we shall invariably find the wants of the former more limited, and supplied with greater facility than the latter. It will appear useless in this place to enter into a train of inquiry respecting the functions and economy of organised beings in general; suffice it to say, that all the varied actions of the animal and vegetable creation may be referable to some physical law: hence it follows, that there is no apparent distinction between the natural and unnatural process of vascular action of the living system, except in degree; for the deposition of the coagulable part of the blood, whether caused by accident or an undue excitement of inflammatory and morbid action, either of the whole or part of the circulating system, or by a rupture of the lymphatic vessels, may, if not timely taken up by the absorbents, be the primary cause of the production of tumor of various appellations; and in this manner, it is presumed, all animal and vegetable monstrosities and irregularities are generated: such, for instance, are warts, corns, wens, nodes, and gallæ quercus. These similarities of unnatural production on plants, most certainly bear a certain relation to the phenomena and growth of tumors on the human body. It is a law, I believe, in the economy of tumors, and worthy of our particular attention, that they rarely continue in an insulated state for any considerable length of time; but, by accelerated arterial action, and tardy disposition of the absorbents, they perpetuate their growth when uninterrupted by surgical aid, as long as the system to which they are connected exists. A tumor, when bearing in itself no appearance of malignity of character in its primary state, if allowed to remain unmolested, will eventually assume a more formidable and alarming aspect; while the patient, on the one hand, from motives of timidity, and the surgeon, on the other, through a mistaken fear of risking his reputation by hazarding an operation, lulls the patient into a state of supinity, and thus they implicitly conspire to deceive each other from day to day, during which time their minds are agitated by a succession of alternate hopes and fears, till the disease, by the slow but inaudible pace of time, becomes exasperated beyond the reach of surgical assistance. This manner of delay I have frequently been a spectator to, and it is in some degree exemplified by the history of the following case.

Mr. Blakey, a respectable schoolmaster of this town, consulted me, about fourteen months ago, respecting a morbid tumor seated on his nose, which had been increasing in size for the space of thirteen years. He informed me, that he had consulted several eminent surgeons on the propriety of its being extirpated, none of whom seemed desirous of undertaking such an unusual operation, lest a fatal hemorrhage might ensue. The tumor extended from the superior part of the nose over the alæ nasi and apex, on both sides, down to the lower part of the upper lip, to such extent that the nostrils and mouth were nearly hermetically closed; and, when laid down to sleep, his breathing was greatly obstructed, unless the tumor was supported by a folding of the pillow placed under it; and likewise, when attempting to drink, it became in part immersed in the liquid, unless it was raised by the hand.

This anomalous tumor designated a vascular aspect, and assumed a deep-red and livid hue, and, from its anatomical structure, it did not exactly correspond in appearance; nor was I able to class it in any distinct species which I had seen described by any author, though it might perhaps partake more of the vascular sarcoma than any other. At the lower part of the tumor, directly under or rather parallel with the septum narium, the cuticular surface of one projecting tubercle was excoriated, from which there was an offensive exudation.

I was

I was informed, that, about six years prior to this period, an irregular surgeon removed a large and troublesome tubercle from near the left alæ, by means of a ligature, which operation proved tedious and painful. On deliberate consideration, and adverting to a similar case related by Mr. Hey, of Leeds, (which is the only operation of this kind that has been published in this kingdom,) I was induced to propose one, as the only resource to effectually eradicate this disgustful wandering of nature. This proposal was willingly assented to, and, on the last day of December, 1811, on the gentleman being seated in a steady chair, and while keeping the natural figure of the nose in my mind, I began the dissection at the superior part of the tumor, as high as the inferior part of the os nasi, continuing the incision along the alæ and apex, on both sides, close to the periosteum and perichondrium, steadily pursuing this project till the whole of the morbid mass was eradicated. The whole of the operation did not occupy much time, nor did the gentleman complain of the least pain. The wound was allowed to bleed spontaneously, and, after the lapse of a few minutes, the hemorrhage ceased, except one artery, which was easily subdued; and this circumstance of no supervening hemorrhage (which had always been dreaded) taking place, together with the success of the operation, I am disposed to attribute solely to the tumor being dissected off so close to the bone and cartilage of the nose. The part was completely healed in three weeks; and now a period of fourteen months has elapsed since the operation, and no traces of the cicatrix can be observed, unless by a very near and minute inspection.

The annexed Plate will furnish the reader with an accurate drawing from life of the size and figure of the tumor prior to its removal; and also of the benefit which Mr. Blakey has derived from the operation.

---

*To the Editors of the Medical and Physical Journal.*

## Figures and Tables

**Figure f1:**